# Alzheimer Classification Using a Minimum Spanning Tree of High-Order Functional Network on fMRI Dataset

**DOI:** 10.3389/fnins.2017.00639

**Published:** 2017-12-01

**Authors:** Hao Guo, Lei Liu, Junjie Chen, Yong Xu, Xiang Jie

**Affiliations:** ^1^Department of Software Engineering, College of Computer Science and Technology, Taiyuan University of Technology, Taiyuan, China; ^2^National Laboratory of Pattern Recognition, Institute of Automation, Chinese Academy of Sciences, Beijing, China; ^3^Department of Psychiatry, First Hospital of Shanxi Medical University, Taiyuan, China

**Keywords:** Alzheimer's disease, fMRI, minimum spanning tree, high-order functional connectivity network, feature selection, classification

## Abstract

Functional magnetic resonance imaging (fMRI) is one of the most useful methods to generate functional connectivity networks of the brain. However, conventional network generation methods ignore dynamic changes of functional connectivity between brain regions. Previous studies proposed constructing high-order functional connectivity networks that consider the time-varying characteristics of functional connectivity, and a clustering method was performed to decrease computational cost. However, random selection of the initial clustering centers and the number of clusters negatively affected classification accuracy, and the network lost neurological interpretability. Here we propose a novel method that introduces the minimum spanning tree method to high-order functional connectivity networks. As an unbiased method, the minimum spanning tree simplifies high-order network structure while preserving its core framework. The dynamic characteristics of time series are not lost with this approach, and the neurological interpretation of the network is guaranteed. Simultaneously, we propose a multi-parameter optimization framework that involves extracting discriminative features from the minimum spanning tree high-order functional connectivity networks. Compared with the conventional methods, our resting-state fMRI classification method based on minimum spanning tree high-order functional connectivity networks greatly improved the diagnostic accuracy for Alzheimer's disease.

## Introduction

In recent years, complex brain network analyses from the whole-brain perspective have become increasingly used to study neuropsychiatric diseases (van Diessen et al., 2013). Complex brain network analysis helps clarify the mechanisms of neuropsychiatric disorders and has the potential to provide relevant imaging markers that may offer new perspectives for the diagnosis and evaluation of clinical brain diseases (Nixon et al., 2014).

Resting-state functional magnetic resonance imaging (rs-fMRI) using blood oxygenation level-dependent (BOLD) signals as neurophysiological indicators can detect spontaneous low-frequency brain activity and has been successfully applied to the diagnosis of Alzheimer's disease (AD) (Sanz-Arigita et al., 2010; Khazaee et al., 2015). Functional connectivity is defined as the “temporal correlations between spatially remote neurophysiological events” (Friston et al., 1993). Unlike anatomical connectivity that describes the physical connections between two brain sites and effective connectivity which characterizes the influence that a neural system may exert over another, functional connectivity examines regional interactions in the brain at a macro level. Commonly, functional connectivity is measured by correlation methods, including linear and non-linear, between BOLD signals of distinct brain regions that has revealed meaningful organization of spontaneous fluctuations in the resting brain. In traditional functional connectivity network analysis, it is assumed that the correlation between different brain regions does not change with time during rs-fMRI scanning. Because these seed-based correlation approaches represent the relationship between two regions of interest as a single correlation coefficient that is calculated from the time series of the entire scan; but, temporal variations in this value will not be captured (Salvador et al., 2005; Achard et al., 2006; Wang et al., 2010; Suk et al., 2013; Zhang et al., 2013). These methods ignore the changes of neural activity or interaction that may occur during the scan.

Given the known dynamic, condition-dependent nature of brain activity^1^, it is natural to expect that functional connectivity metrics computed on fMRI data will exhibit variation over time. As recent studies both on animals and humans have highlighted the non-stationary nature of functional connectivity in BOLD fMRI data (Chang and Glover, 2010; Hutchison et al., 2013). Recent studies have suggested that brain functional connectivity is characterized by abundant temporal information (Chang et al., 2013; Leonardi et al., 2013; Allen et al., 2014; Damaraju et al., 2014; Tomasi et al., 2014; Calamante et al., 2017). Whether in a resting or tasking state, the functional connectivity changes with the time pattern of neural activity (Hutchison et al., 2013; Tomasi et al., 2014).

Dynamic changes in neural interactions may affect the topological structure and associated intensity of the temporally related functional connectivity, and these subtle and transient changes may be caused by disease (Chang et al., 2013; Hutchison et al., 2013; Leonardi et al., 2013; Allen et al., 2014; Damaraju et al., 2014; Tomasi et al., 2014). Damaraju et al. (2014) analyzed patients with schizophrenia using the static functional connectivity based on the entire time series and the dynamic functional connectivity based on sliding windows, and the results showed that dynamic analysis could deepen our understanding of brain activity in schizophrenia. Leonardi et al. (2013) assumed that dynamic functional connectivity could provide more information about brain organization. Moreover, Wee et al. (2016) used the sliding window method to divide the entire rs-fMRI time series and established functional connectivity networks of the whole brain. Using dynamic functional connectivity analysis, they found abundant abnormal features for the diagnosis of mild cognitive impairment (MCI) and constructed a classification method based on a sparse temporal dynamic network. Increasing evidence shows that functional connectivity change dynamically in the resting state, and these dynamic functional connectivity reflect important information. Rubinov et al. (Rubinov and Sporns, 2011) applied to graphical representations of functional connectivity with sliding window approach. The authors reported differences in the “dwell time” within different sub-network configurations of the default mode network between Alzheimer's patients and age-matched healthy controls. With the same method, Quevenco et al. (2017) found that altered dynamic anterior-posterior brain connectivity was a characteristic of low memory performance and one of the important features in AD discrimination.

Chen et al. (2016) used the sliding window to divide the whole rs-fMRI time series, built a functional connectivity network in each time window, stacked all the networks, and used a clustering algorithm to divide all relevant time series into several clusters. The average time series of each cluster was then taken as a new node, and the Pearson correlation coefficient between each node pair was calculated as the weight of the connectivity. In this way, high-order functional connectivity networks were constructed, and dynamic functional connectivity analysis took the time-varying characteristic into account. However, a clustering method was employed to decrease the associated computational costs, and the randomness of the selection of initial clustering centers and the number of clusters greatly influenced the classification accuracy. At the same time, the time series of all connectivity within each cluster were averaged, so that the network lost neurological interpretability.

The minimum spanning tree (MST) (Vikas, 2010) is one of the classic methods in graph theory that can obtain the general information and index structure of the graph and remove redundant information. Lee et al. (2006) was the first to apply a method using the MST to analyze brain networks. This unbiased method greatly simplifies the network structure but preserves its core framework, which avoids the influences of network sparseness and other parameters on network structure. It also guarantees the neurological interpretability of the network and has been widely used in neuroimaging. Recently, the MST has been applied in psychiatric studies (Tewarie et al., 2014; van Dellen et al., 2014). The edges in the network are simplified with this method, which ensures that the selected spanning tree has the smallest possible weight.

In the current study, the MST was used to construct a high-order functional connectivity network to simplify the structure while preserving its core framework. We also introduce the Relief feature selection method based on pairwise redundancy analysis and the multi-parameter optimization framework for feature selection and classifier construction. The MST high-order functional connectivity network (HON-MST) can reveal higher-level and more complex interactive information than conventional functional connectivity networks. Importantly, HON-MSTs are derived from low-order functional connectivity networks (the networks who constructed by the traditional seed-based correlation approaches), which does not affect the analysis of different subjects and also helps identify more accurate AD biomarkers. Compared with the conventional method, the rs-fMRI classification method based on HON-MST greatly improved the diagnostic accuracy of AD.

## Materials and methods

### Proposed framework

**Figure 1:**
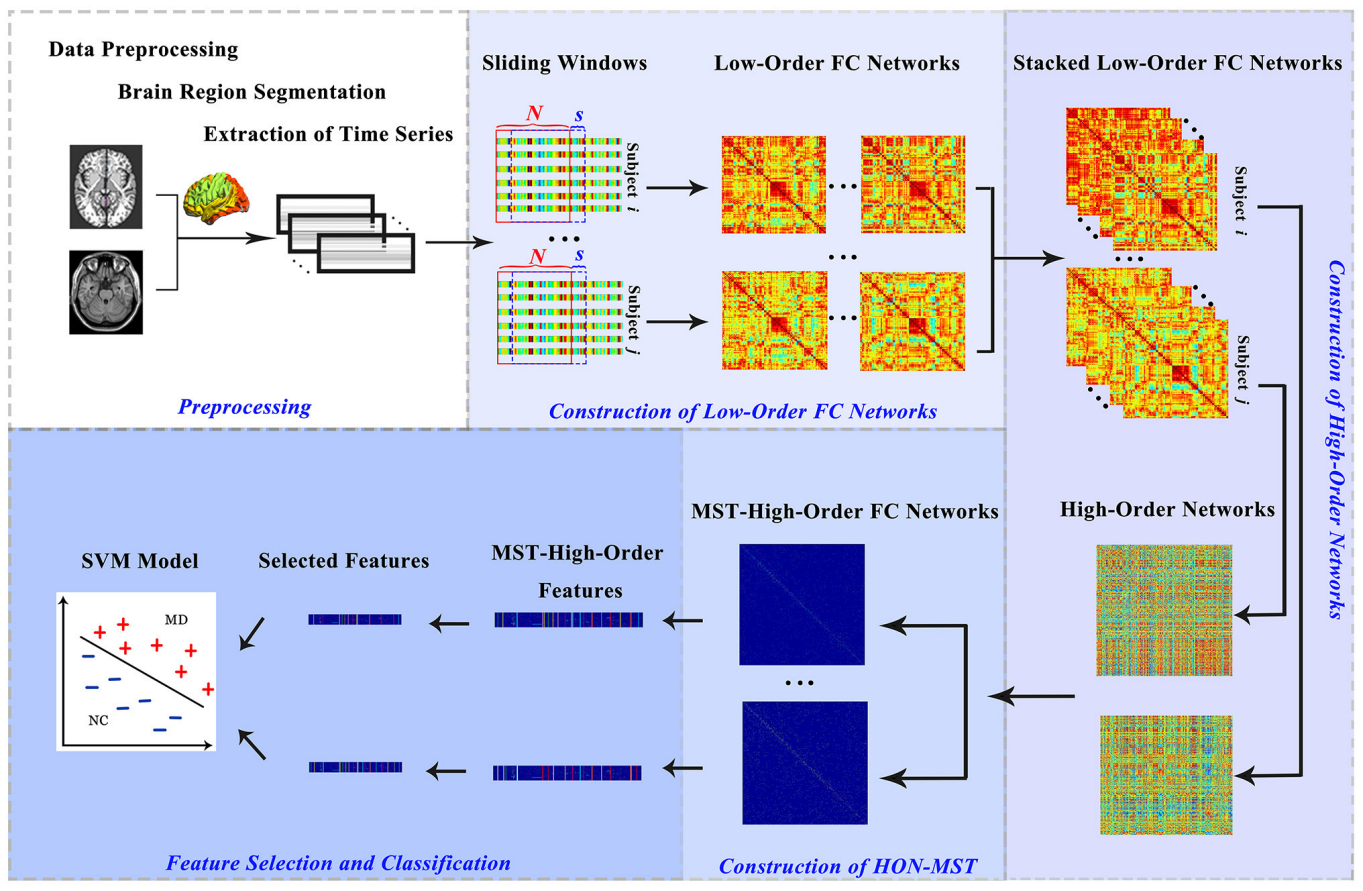
Construction and classification process of HON-MSTs. FC, functional connectivity; SVM, support vector machine.

The data classification methods of rs-fMRI based on HON-MST usually include data preprocessing, construction of low-order and high-order functional connectivity networks, HON-MST construction, feature selection, and classification. Specifically, the framework consists of the following five steps (Figure 1):

1. Data acquisition and preprocessing.
2. Constructing low-order functional connectivity networks.
  2.1 Selecting a fixed sliding window to segment the average time series of each brain region.
  2.2 Using the Pearson correlation approach, calculating the degree of correlation of the average time series of each region under each time window and obtaining the low-order functional connectivity matrices.
3. Constructing high-order networks.
  3.1 Stacking all low-order functional connectivity matrices, i.e., extracting the values of the corresponding elements in the low-order functional connectivity matrices of each time window.
  3.2 Constructing a high-order network by calculating the Pearson correlation coefficient between each pair for the entire time series.
4. HON-MST construction.
  This high-order network is pruned by the MST method to construct the HON-MST.
5. Feature selection and classification model construction.
  5.1 Calculating weighted-graph local clustering coefficients (Rubinov and Sporns, 2010) for each node.
  5.2 Using the multi-parameter optimization framework, defining the weighted-graph local clustering coefficients of each node as classification features, and constructing the classifier.
  5.3 Using the cross-validation method to test the constructed classifiers and obtain the final classification results.

In addition, to compare the effect of network pruning using the MST method, after the high-order networks are obtained, they are filtered according to the statistical significance of the connectivity to construct high-order functional connectivity networks. The feature selection and classification models are constructed according to the fifth step to identify differences in the classification results obtained by the two methods.

### Data acquisition and preprocessing

This study was approved by the medical ethics committee of Shanxi Province (approved certification number 2012013). Twenty-eight healthy right-handed volunteers and 38 subjects with AD underwent rs-fMRI in a 3T MR scanner (Trio 3-Tesla scanner; Siemens, Erlangen, Germany). The subjects' demographics and clinical characteristics are shown in Table 1. All AD patients underwent a complete physical and neurological examination, standard laboratory tests, and an extensive battery of neuropsychological assessments. All AD patients met the criteria for a diagnosis of probable AD according to the National Institute on Aging Alzheimer's Association guidelines (McKhann et al., 2011). Data collection was performed by radiologists familiar with MRI at the First Hospital of Shanxi Medical University. During the scans, participants were asked to relax, close their eyes, and stay awake. The parameter settings were as follows: 33 axial slices, repetition time (TR) = 2000 ms, echo time (TE) = 30 ms, thickness/skip = 4/0 mm, field of view (FOV) = 192 × 192 mm, matrix = 64 × 64 mm, flip angle = 90°, and 248 volumes. The first 10 volumes of time series were discarded for magnetization stabilization. See Supplemental Text S1 for details on the scanning parameters.

**Table 1:** Demographics and clinical characteristics of the subjects.

	**NC**	**AD**	***P*-value**
Age	72.6 ± 3.42	71.4 ± 4.68	0.44^a^
Gender(Male/Female)	13/15	15/23	0.57^b^
Handedness (Right/Left)	28/0	38/0	
MMSE	26.1 ± 3.2	22.8 ± 2.1	<0.0001^a^

*Values are mean ± standard deviation; AD, Alzheimer's disease; NC, normal controls; MMSE, Mini-Mental State Examination*.

a *Two-sample t-test*.

b *Pearson Chi-square test*.

Image preprocessing was carried out using SPM8 software (http://www.fil.ion.ucl.ac.uk/spm). First, slice-timing correction and head-movement correction were carried out. Two samples exhibiting more than 3.0 mm of translation and 3.0° of rotation were discarded (Bansal et al., 2009; Abrams et al., 2013; Wilke, 2014). Then, the images were subjected to 12-dimensionally optimized affine transformation, which was normalized to the Montreal Neurological Institute (MNI) standard voxel space of 3 × 3 × 3 mm. Finally, linear detrending and band-pass filtering (0.01–0.10 Hz) were performed to reduce low-frequency drift and high-frequency physiological noise.

### Low-order functional connectivity network construction

The whole brain was divided into 90 regions (45 per hemisphere) using an automated anatomical labeling (AAL) template (Tzourio-Mazoyer et al., 2002). For the AAL template, the whole brain is divided into 116 regions, but only 90 are considered by excluding the cerebellum (see Supplemental Text S2 for brain region names and abbreviations). Each regional mean time series was regressed against the average cerebral spinal fluid (CSF) and white matter signals, as well as the six parameters from motion correction. The arithmetic mean of the BOLD signals of all voxels included in each brain region was calculated to represent the signal value of the node. The BOLD signals of all voxels included in each brain region were extracted at different time points and averaged to obtain the average time series of brain regions.

Next, a sliding window with a fixed length was selected, and the average time series extracted from each brain area was segmented by time window according to a fixed step size (see Supplemental Figure S1 for the time window division diagram and Supplemental Text S3 for an illustration of the dynamical variations of the functional connectivity strength at different time windows). Suppose the regional mean rs-fMRI time series associated with the *i*-th region of interest (ROI) of the *l*-th subject is expressed as *xi*^(*l*)^, then, *xi*^(*l*)^can be divided into *K* overlapping parts, where the value of *K* is given by the following formula:

(1) $$\begin{array}{r} K=\left\lfloor \left(M-N\right)/S+1\right\rfloor . \end{array}$$

Here, *M* represents the *xi*^(*l*)^ length, *N* represents the length of the sliding window, and *S* represents the step size of each sliding window. The interval of step size is set as 1 TR. In processing, TR is 2 s so one step is 2 s. In the *K* parts, each part is represented by *xi*^(*l*)^(*k*)1 ≤ *k* ≤ *K*, which represents the rs-fMRI time series in a relatively short period of time.

The *k*-th part of all the *R* brain regions of the *l*-th subject can be expressed as a matrix $X^{\left(l\right)}\left(k\right)=\left[{x_{1}}^{\left(l\right)}\left(k\right),{x_{2}}^{\left(l\right)}\left(k\right),\cdots \;,{x_{R}}^{\left(l\right)}\left(k\right)\right]\in R^{N\times R}$, where *R* represents a total of *R* brain regions. For each of these *R* sequences, the pairwise correlation degree was calculated, and a temporal functional connectivity matrix consisting of the *k*-th part of all *R* brain regions of the *l*-th subject can be obtained. The degree of correlation between *xi*^(*l*)^(*k*) and *xj*^(*l*)^(*k*) was given by the following equation:

(2) $$\begin{array}{r} Cij^{\left(l\right)}\left(k\right)=corr\left(xi^{\left(l\right)}\left(k\right),xj^{\left(l\right)}\left(k\right)\right). \end{array}$$

Here, *xi*^(*l*)^(*k*) represents the *k*-th part of the *i*-th brain region of the *l*-th subject, and *xj*^(*l*)^(*k*) represents the *k*-th part of the *j*-th brain region of the *l*-th subject. Taking $\left\{{x_{i}}^{\left(l\right)}\left(k\right)\right\}$ as vertices and {*Cij*^(*l*)^(*k*)} as the weights of edges for each subject, *K* temporal low-order functional connectivity networks can be established. Here, {*Cij*^(*l*)^(*k*)} is called low-order functional connectivity. The *k*-th temporal low-order functional connectivity network of the *l*-th subject is represented by *G*^(*l*)^(*k*), which reflects the change in connectivity intensity between all brain regions over time.

Since *K* temporal low-order functional connectivity networks were established for each subject, all ${C_{ij}}^{\left(l\right)}\left(k\right)$ can be combined for the connection (*i, j*) between each pair of brain regions of the *l*-th subject, and a new correlation time series can be obtained: ${y_{ij}}^{\left(l\right)}=\left[{C_{ij}}^{\left(l\right)}\left(1\right),{C_{ij}}^{\left(l\right)}\left(2\right),\cdots \;,{C_{ij}}^{\left(l\right)}\left(K\right)\right]\in R^{K}$. Considering correlation coefficient matrix symmetry, the total number of correlation time series $\left\{{y_{ij}}^{\left(l\right)}|1\leq i\leq R-1,i+1\leq j\leq R\right\}$ was [*R*(*R* − 1)]/2. It should be emphasized that the correlation time series ${y_{ij}}^{\left(l\right)}$ obtained is different from the average time series of each brain region extracted in the first step. The former reflects the time-dependent nature of functional connectivity over time, while the latter only records changes in the mean BOLD signal for each ROI during the rs-fMRI scan. In summary, the correlation time series ${y_{ij}}^{\left(l\right)}$ reflects dynamic functional connectivity with abundant temporal properties, which may be due to the effect of dynamic neural interaction changes on functional connectivity strength. It is therefore possible to reveal temporal variation of functional connectivity between different brain regions and produce more detailed interactive information.

### Construction of high-order functional connectivity network

The main purpose of this paper was to reveal the intrinsic relationship between the relevant time series $\left\{y_{ij}^{\left(l\right)}\right\}$ and the abundant temporal properties it contains, so the Pearson correlation coefficient was also calculated between each pair of correlated time series for each subject, in which this correlation coefficient between a pair of correlated time series $\left\{y_{ij}^{\left(l\right)}\right\}$ and $\left\{y_{pq}^{\left(l\right)}\right\}$ of the *l*-th subject can be expressed as:

(3) $$\begin{array}{r} H_{ij,pq}^{\left(l\right)}=corr\left(y_{ij}^{\left(l\right)},y_{pq}^{\left(l\right)}\right). \end{array}$$

Here, $\left\{y_{ij}^{\left(l\right)}\right\}$ is the dynamic functional connectivity between the *i*-th and *j*-th brain regions of the *l*-th subject, and $\left\{y_{pq}^{\left(l\right)}\right\}$ is the dynamic functional connectivity between the *p*-th brain region and the *q*-th brain region of the *l*-th subject. $H_{ij,pq}^{\left(l\right)}$, the high-order correlation, indicates functional connectivity between the *i*-th and *j*-th brain regions of the *l*-th subject and the degree of functional association between the *p*-th and *q*-th brain regions. It reflects the impact of a functional connectivity on the strength of other functional connectivity. It describes a more complex and abstract interaction pattern, reflects the interaction of up to four brain regions, and reveals more brain regions with time changes in the interaction of more detailed information. In other words, constructing high-order functional connectivity considers the characteristics of time-varying features and describes more interactive information between more brain regions compared to the conventional approach. Thus, a new network could be constructed by taking $\left\{y_{ij}^{\left(l\right)}\right\}$ as new vertices and $\left\{H_{ij,pq}^{\left(l\right)}\right\}$ as the weights of new edges between node $\left\{y_{ij}^{\left(l\right)}\right\}$ and node $\left\{y_{pq}^{\left(l\right)}\right\}$.

In the conventional approach of brain network construction, the strength of connectivity between two brain regions is indicated by the value of correlation coefficient. When it reaches a certain threshold, the regions are considered to have functional connectivity. At the same time, the correlation coefficient reflects functional connectivity strength. Therefore, it is necessary to filter the connectivity of the new network, remove edges that are not significant, and maintain edges that are statistically significant (*P* < 0.05, false-discovery rate method-corrected, seven comparisons). Significant high-order correlations were termed high-order functional connectivity $\left\{{\overset{-}{H}}_{ij,pq}^{\left(l\right)}\right\}$. Finally, the high-order functional connectivity networks $G_{H}^{\left(l\right)}=\left(\left\{y_{ij}^{\left(l\right)}\right\},\left\{{\overset{-}{H}}_{ij,pq}^{\left(l\right)}\right\}\right)$ could be obtained.

### Construction of the MST high-order functional connectivity network

Using the MST method, all nodes were reserved, the edges were pruned, and the trees with the smallest total weight among all spanning trees were obtained. With $\left\{y_{ij}^{\left(l\right)}\right\}$ as the node, and $\left\{H_{ij,pq}^{\left(l\right)}\right\}$ as the weight between node $\left\{y_{ij}^{\left(l\right)}\right\}$ and node $\left\{y_{pq}^{\left(l\right)}\right\}$, a new undirected weighted functional connectivity network was constructed.

To prune the network and improve its performance, the MST method based on the Kruskal algorithm was used to search for the MST in undirected weighted networks and remove useless and redundant edges. The network was simplified, but the impact of the larger edges on the network performance was retained. Thus, a HON-MST could be established (see Supplemental Text S4 for details of the algorithm).

### Feature selection and classification

#### Feature definition

After constructing the functional connectivity networks, the weighted-graph local clustering coefficient (Rubinov and Sporns, 2010) was defined as the feature, and then the weighted-graph local clustering coefficients of each node in the HON-MST were calculated. The weighted-graph local clustering coefficients represent the degree of node aggregation in complex networks. This indicator better reflects the prevalence of cluster connectivity around individual nodes (Rubinov and Sporns, 2010), which were widely used in previous studies (Chen et al., 2016). The mathematical definition of weighted-graph local clustering coefficients is as follows:

(4) $$\begin{array}{r} f_{i}=\frac{2\sum_{j:j\in \Delta i}{\left(w_{ij}\right)}^{\frac{1}{3}}}{|\Delta_{i}|\left(|\Delta_{i}|-1\right)} \end{array}$$

where Δ_*i*_ represents the set of vertices directly connected to node *i*, |Δ_*i*_| represents the number of all vertices connected to node *i*, and *w*_*ij*_ denotes the weight of the edge connecting nodes *i* and *j*.

Multiple linear regression analyses were applied to remove the confounding effects of age, gender, and education level (independent variable: the area under the curve [AUC] of each network property; dependent variables: age, gender, and educational attainment). The results did not reveal significant correlation between the weighted-graph local clustering coefficient and confounding variables (see Supplemental Table S1 for detailed results).

Feature selection involves choosing the most representative optimal feature sets from a set of features. The feature vector extracted from the functional connectivity network may contain some features that are not related to or redundant for the diagnosis of AD. A useful tool in this context is the Relief algorithm, which was first proposed by Kira (Kira and Rendell, 1992) and was primarily used to classify two types of data. This method is suitable for classifying patients with AD and normal subjects. To reduce the influence of irrelevant or redundant features and improve generalization performance, we used the Relief feature selection method to obtain the weight of each feature according to the correlation of each feature and category, and then the features were filtered according to the set threshold, thereby obtaining a new feature set. Since the Relief feature selection method could not remove the redundant features, the extracted feature sets were thus subjected to by pairwise redundancy analysis. By calculating Pearson correlation coefficients, the features with low weighting were removed to obtain the final feature set. In this way, the key features associated with AD could be identified.

#### Multi-parameter optimization framework

**Figure 2:**
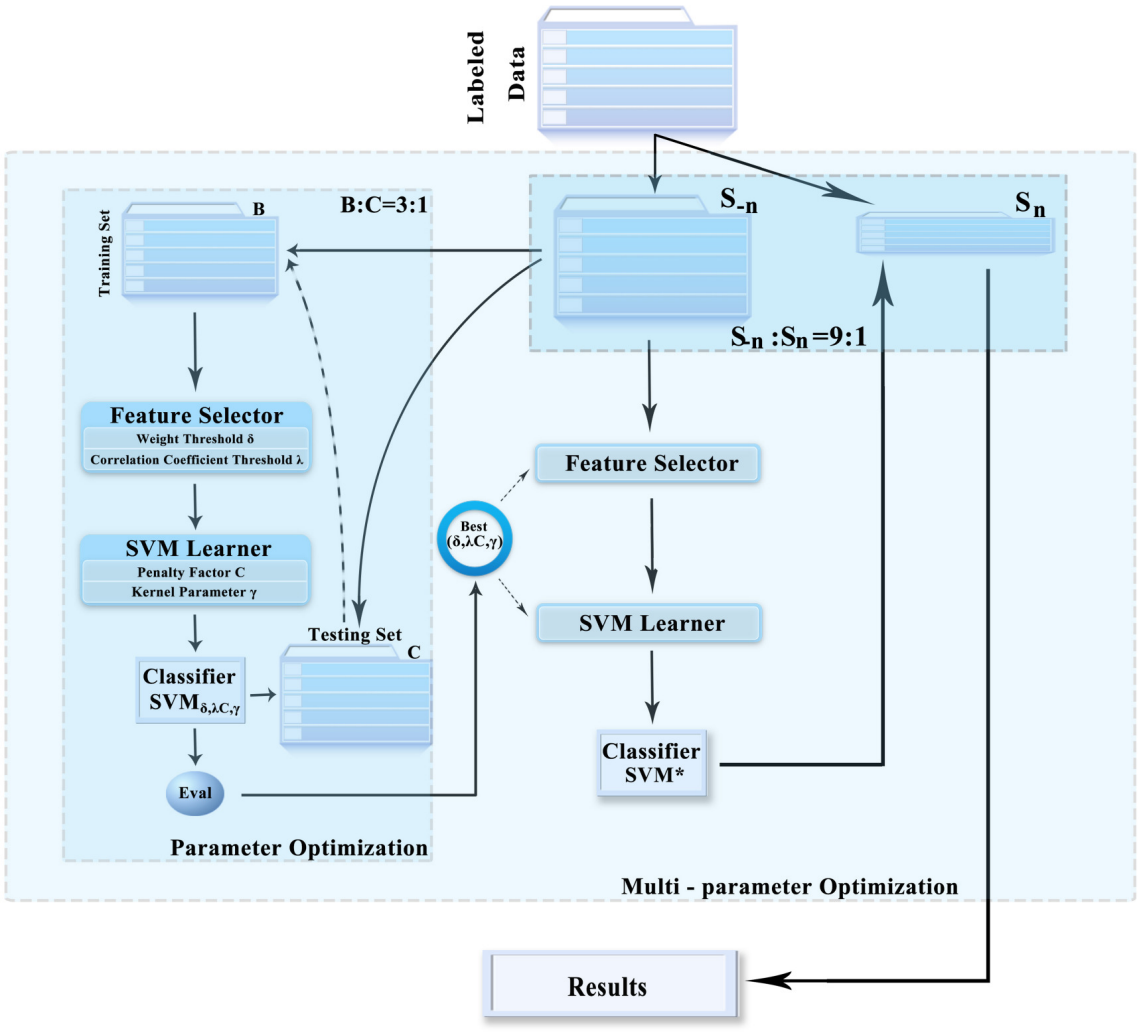
Multi-parameter optimization framework. SVM, support vector machine.

This paper prevents a multi-parameter optimization framework that obtains the optimal combination of parameters. It can prevent overfitting and improve the generalization performance of the classifier, which makes feature selection and classification more accurate and effective. The feature selection method, classifier, and framework are illustrated in Figure 2. It includes the following three steps:

1. The entire datasets were randomly divided into 10 parts; 1 of these was used as the test set (*S*_*n*_), and the other 9 were used as the training set (*S*_−*n*_). Then, the input dataset *S*_−*n*_ was divided into 2 groups (training set *B* and test set *C*) at a ratio of about 3:1.
2. In training set *B* obtained in step 1, the different feature selection parameters and the support vector machine (SVM) parameter combination were selected to construct the classifier. Based on the performance of each set of parameters in test set *C*, the optimal combination of parameters was obtained.
3. The entire datasets were randomly divided into 10 parts; 1 of these was used as the test set (*S*_*n*_), and the other 9 were used as the training set (*S*_−*n*_). Then, the input dataset *S*_−*n*_ was divided into 2 groups, that is, training set *B* and test set *C*, at a ratio of about 3:1.

#### Classification

SVM transforms the original data into a high-dimensional feature space by nonlinear change and seeks a hyperplane that maximizes the interval between classes, separating the samples of one class from those of the others. It has a unique advantage in dealing with high-dimensional, nonlinear, and small sample data. In the multi-parameter optimization framework, we used the LIBSVM toolkit (Chang and Lin, 2011) (http://www.csie.ntu.edu.tw/~cjlin/libsvm/) based on MATLAB.

The 10-fold cross-validation method (Chang and Lin, 2011) was used to evaluate the generalization performance of the classifier. Specifically, the subjects were randomly divided into 10 parts; 1 of these was used as the test set *S*_*n*(*n* = *1, 2*……*10*)_, and the other 9 were used as the training set *S*_−*n*(*n* = *1, 2*……*10*)_. Among them, the SVM classifiers were constructed on the training set using the multi-parameter optimization framework, taking the mean of the 10 results to assess classifier performance. At the same time, 10-fold cross-validation that was repeated 100 times was carried out to obtain more accurate results.

## Results

### Discriminative functional connectivity and brain regions

**Figure 3:**
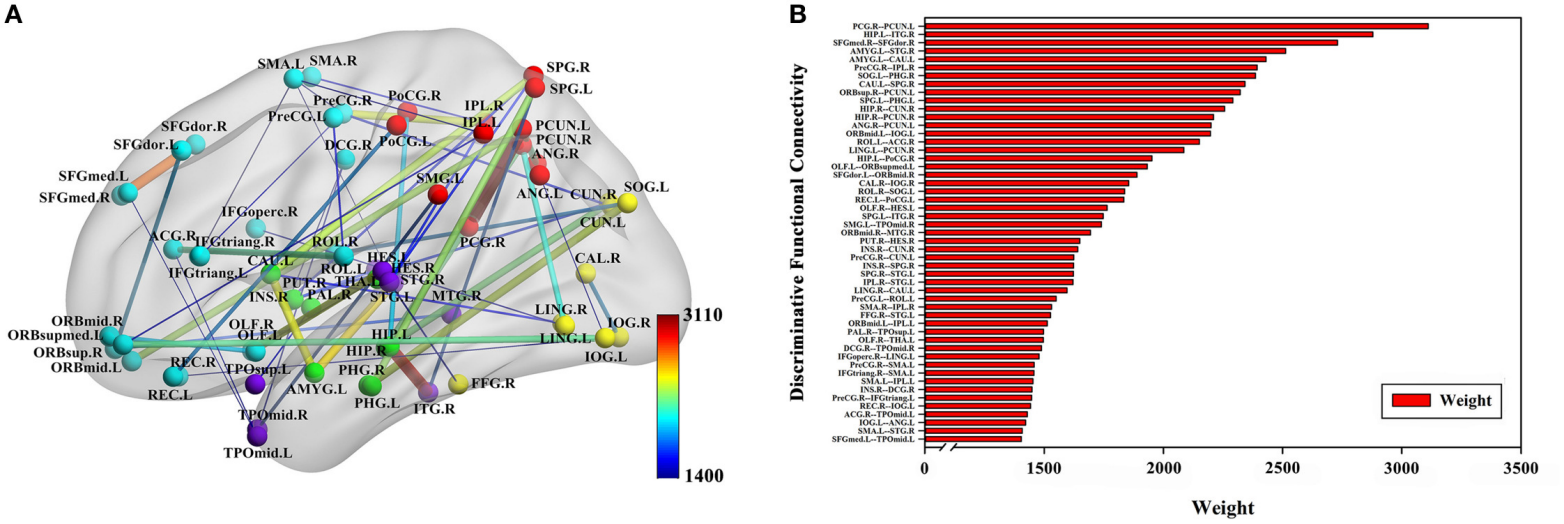
Fifty-one discriminative functional connectivity are selected by feature selection. **(A)** The distribution of 51 discriminative functional connectivity in the brain. **(B)** All discriminative functional connectivity weight graphs. The color of the node in **(A)** represents the module to which the node belongs. Blue represents frontal, yellow represents occipital, red represents parietal, green represents subcortical and purple represents temporal. The color and thickness of the connectivity represent the weight of the connectivity. The weight refers to the weight value assigned to each feature in the Relief feature selection method.

**Figure 4:**
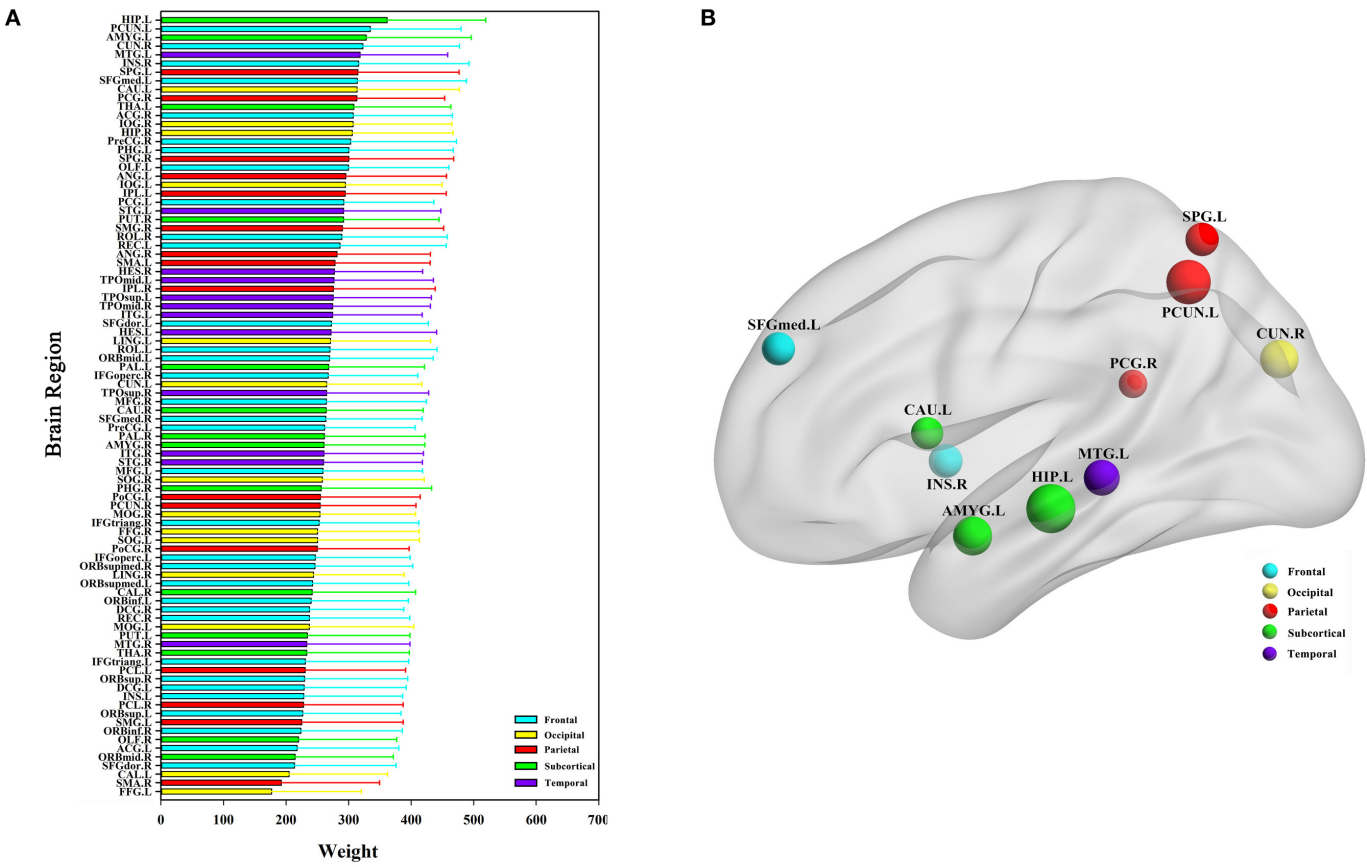
Abnormal brain regions. **(A)** All brain regions weights graph. **(B)** 10 abnormal brain regions whose weight values are greatest. **(B)** Selects the largest weight of the 10 abnormal brain regions for further analysis, and the size of the nodes represents the weight values of the nodes. The weight value in the figure is the average weight of all functional connectivity connected to each brain region.

The classification method based on the HON-MST had 51 discriminative functional connectivity (Table 2). A comprehensive and detailed analysis of the results from the brain regions, functional connectivity, and other aspects of the analysis is presented. Figure 3 shows the 51 connectivity selected by feature selection and their weights (each feature was assigned a different weight with the Relief feature selection algorithm). Among them, the largest functional connectivity weight was between the left precuneus and right posterior cingulate gyrus. The distribution of these 51 functional connectivity in the brain is shown in Figure 3A. Figure 3B shows the weights of all functional connectivity. Figure 4 was generated to identify which brain regions can discriminate AD patients based on the major discriminatory brain regions and their mean weights. The average weight of each region is the average of the weights of all its functional connectivity. According to the weight value of each feature (i.e., each functional connectivity), the average weight of all functional connectivity involved in each brain area is shown in Figure 4A. These weight values were sorted to identify abnormal brain regions, and some of the higher weighted regions were selected for the key analysis.

**Table 2:** Discriminative functional connectivity.

**Functional Connectivity**		**Properties**		
**ROI A**	**ROI B**	**Weights**	**Ave. CC in ADs**	**Ave. CC in NCs**
PCUN.L	PCG.R	3,110.48	1.25	1.23
HIP.L	ITG.R	2,878.38	1.28	1.26
SFGmed.R	SFGdor.R	2,730.09	0.63	0.62
AMYG.L	STG.R	2,512.89	0.61	0.63
AMYG.L	CAU.L	2,430.84	1.32	1.30
IPL.R	PreCG.R	2,393.80	0.35	0.34
SOG.L	PHG.R	2,386.57	1.06	1.05
CAU.L	SPG.R	2,342.50	0.71	0.72
PCUN.L	ORBsup.R	2,322.26	0.76	0.74
SPG.L	PHG.L	2,292.41	0.33	0.30
CUN.R	HIP.R	2,256.38	0.71	0.73
PCUN.R	HIP.R	2,210.56	1.10	1.11
PCUN.L	ANG.R	2,200.40	0.81	0.80
ORBmid.L	IOG.L	2,197.00	1.17	1.15
ROL.L	ACG.R	2,150.74	1.08	1.07
LING.L	PCUN.R	2,086.05	0.75	0.71
HIP.L	PoCG.R	1,951.98	1.17	1.18
OLF.L	ORBsupmed.L	1,932.64	1.15	1.15
SFGdor.L	ORBmid.R	1,889.15	0.60	0.60
CAL.R	IOG.R	1,854.08	1.18	1.16
SOG.L	ROL.R	1,837.50	0.73	0.73
REC.L	PoCG.L	1,834.27	0.59	0.58
HES.L	OLF.R	1,763.97	0.80	0.80
SPG.L	ITG.R	1,747.43	0.40	0.41
SMG.L	TPOmid.R	1,740.55	0.53	0.51
ORBmid.R	MTG.R	1,694.61	1.30	1.30
PUT.R	HES.R	1,694.25	0.41	0.40
INS.R	CUN.R	1,640.92	0.91	0.90
CUN.L	PreCG.R	1,623.96	0.61	0.59
INS.R	SPG.R	1,623.64	0.69	0.67
STG.L	SPG.R	1,622.53	1.06	1.05
STG.L	IPL.R	1,621.21	0.67	0.68
CAU.L	LING.R	1,596.39	0.83	0.81
PreCG.L	ROL.L	1,550.66	0.51	0.50
SMA.R	IPL.R	1,530.66	0.63	0.62
STG.L	FFG.R	1,526.44	1.14	1.14
ORBmid.L	IPL.L	1,513.33	0.41	0.40
TPOsup.L	PAL.R	1,496.92	0.62	0.61
THA.L	OLF.R	1,496.23	1.04	1.04
DCG.R	TPOmid.R	1,488.46	1.18	1.16
LING.L	IFGoperc.R	1,478.87	0.90	0.90
SMA.L	PreCG.R	1,458.01	0.54	0.54
SMA.L	IFGtriang.R	1,457.45	0.94	0.92
SMA.L	IPL.L	1,453.08	0.62	0.62
INS.R	DCG.R	1,448.98	1.09	1.08
PreCG.R	IFGtriang.L	1,447.17	0.65	0.65
IOG.L	REC.R	1,442.83	0.51	0.50
TPOmid.L	ACG.R	1,429.63	1.15	1.11
IOG.L	ANG.L	1,422.53	1.14	1.10
SMA.L	STG.R	1,408.45	1.13	1.16
SFGmed.L	TPOmid.L	1,403.93	0.53	0.51

*Weight refers to the weight value assigned to each feature in the Relief feature selection algorithm. Ave. CC in ADs refers to the average local clustering coefficient (see formula (4)) of each node in AD group. Ave. CC in NCs refers to the average local clustering coefficient of each node in NC group. L, the left brain region; R, the right brain region*.

**Figure 5:**
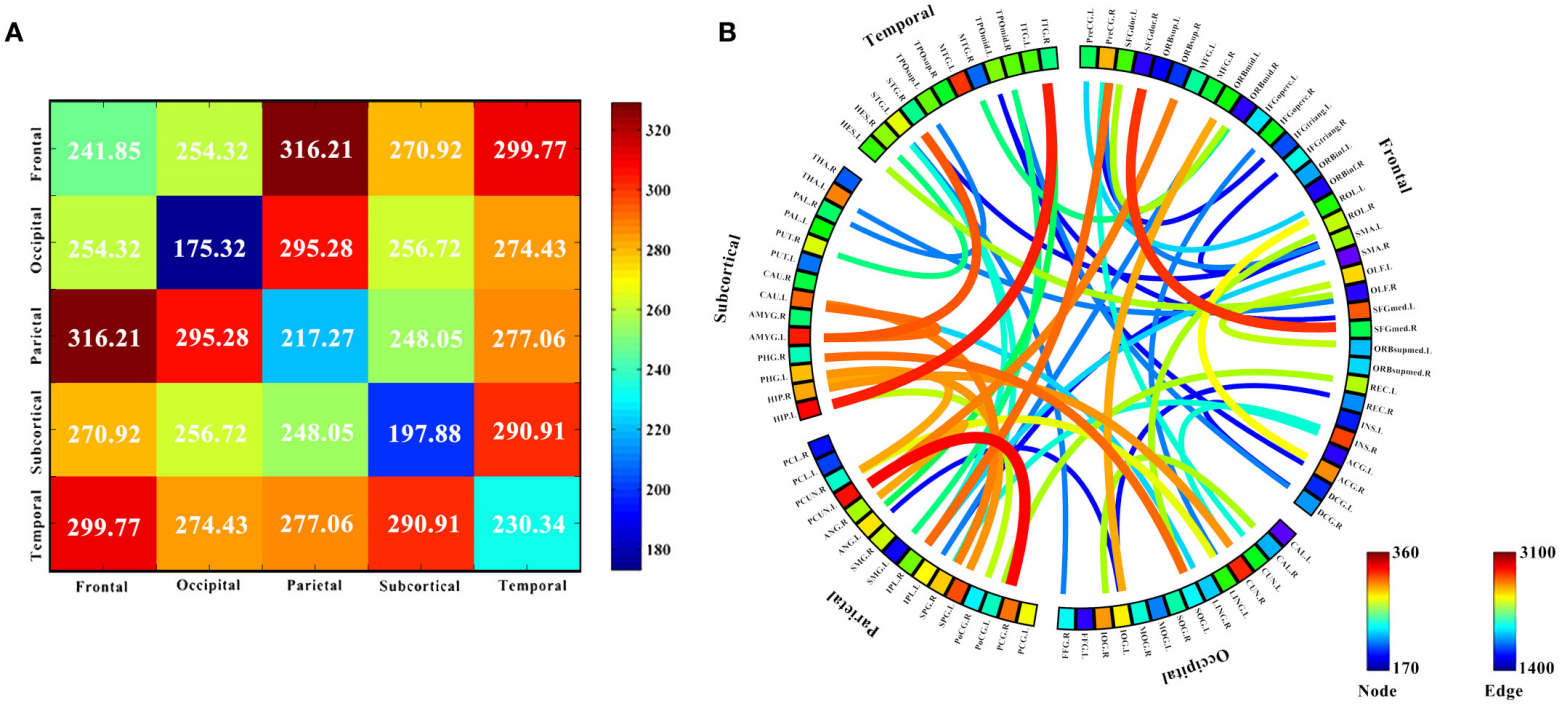
Analysis of the interaction between the five modules. **(A)** The interaction matrix between different brain modules. **(B)** Modular analysis 51 discriminative functional connectivity. The 90 brain regions were divided into 5 modules: frontal, occipital, parietal, subcortical and temporal, and discriminative interaction information between five different brain modules was analyzed. **(A)** Shows the interaction matrix between the five modules, that is, the average of the functional connectivity weights between all the brain regions in one module and all the brain regions in the other modules. The color of the circle in **(B)** represents the average weight of all functional connectivity to which each brain region is connected. The color and thickness of the connectivity line indicate the weight value of each functional connectivity.

Ten discriminatory brain regions with the largest weights (descending order) were selected: the left hippocampus, left precuneus, left amygdala, right cuneus, left middle temporal gyrus, right insula, left superior parietal gyrus, left superior frontal gyrus, medial, left caudate nucleus, and right posterior cingulate gyrus, where the weight of the left hippocampus was significantly higher than that of other brain regions. To analyze the abnormal interactions between different modules, the 90 brain regions were divided into 5 modules: frontal, occipital, parietal, subcortical, and temporal (Mears and Pollard, 2016). Figure 5 shows the abnormal interactions between these modules. Figure 5A shows the interaction matrix between two modules, that is, the average of the functional connectivity weights between all of the brain regions in one module and all of the brain regions in the other modules. The interaction weight between the frontal and parietal, frontal and temporal, and parietal and occipital modules were significantly higher than those of other modules. Figure 5B shows the distribution of the 51 discriminative functional connectivity in these 5 modules; those with greater weights mainly connect the temporal and subcutaneous modules and the frontal and parietal modules.

### Classification results

The high-order functional connectivity network and the HON-MST were constructed using the same subjects' data. The weighted local clustering coefficient of the node was defined as the feature, while the multi-parameter optimization framework was used for feature selection and classification. The accuracy, specificity, and sensitivity of the rs-fMRI classification method based on the HON-MST were 98.16, 96.68, and 98.92%, respectively (Table 3). The results showed that the rs-fMRI classification method of AD based on the HON-MST could accurately distinguish between control and AD subjects.

**Table 3:** Classification results for different functional connectivity networks.

**Method**	**Research**	**Disease**	**Accuracy (%)**	**Specificity (%)**	**Sensitivity (%)**
PAN	Guo et al., 2012	MDD	83.00	–	–
	Rosa et al., 2015	MDD	48.33	53.33	43.33
	Wee et al., 2016	eMCI	62.71	60.00	65.52
	This study	AD	63.06	50.56	87.37
PEN	Yu et al., 2013	MDD	84.20	–	–
	Chen et al., 2011	AD	82.00	80.00	85.00
	Wee et al., 2016	eMCI	66.10	76.67	55.17
	This study	AD	66.67	46.43	81.58
HON	Chen et al., 2016	eMCI	88.14	90.00	86.21
	This study	AD	92.51	88.51	93.19
HON-MST	This study	AD	98.16	96.68	98.92

*AD, Alzheimer's disease; eMCI, early mild cognitive impairment; MDD, Major Depressive Disorder; PAN, partial correlation functional connectivity network. PEN, pearson correlation functional connectivity network. HON, high-order functional connectivity network. HON-MST, minimum spanning tree high-order functional connectivity network*.

## Discussion

### Discriminative functional connectivity and brain regions

The weighted-graph local clustering coefficients of HON-MSTs were calculated. A total of 51 discriminative functional connectivity were obtained by the Relief feature selection approach with pairwise redundancy analysis. Among them, the largest functional connectivity weight was between the left precuneus and right posterior cingulate gyrus. These discriminative functional connectivity are important for the diagnosis of AD and are the same as those obtained in previous studies. For example, Toussaint et al. (2014) investigated functional connectivity within the default mode network in normal subjects and AD using rs-fMRI. They found that the functional connectivity between the left precuneus and right posterior cingulate gyrus was an important biomarker for distinguishing AD and normal subjects. In addition, Kim et al. (Kim and Pan, 2015) proposed two highly adaptive tests for group differences in functional connectivity between patients with AD and normal subjects. They found that functional connectivity between the right superior frontal gyrus, dorsolateral and the right superior frontal gyrus, medial was significantly different between the AD and control groups.

In the 51 discriminative functional connectivity, the weights of functional connectivity related to the hippocampus and amygdala were greater. The hippocampus is the core region of atrophy in AD and is associated with episodic memory deficits. Previous studies have found a number of discriminative functional connectivity associated with the hippocampus, including between the left hippocampus and right inferior temporal gyrus (Wang et al., 2006), the right hippocampus and right precuneus (Kim et al., 2012), and the right hippocampus and right cuneus (Zhou et al., 2015). For example, Wang et al. (2006) used rs-fMRI to examine hippocampal connectivity changes comparing 13 patients with mild AD and 13 healthy age-matched controls. They found that functional connectivity between the left hippocampus and right inferior temporal gyrus was significantly different between patients with AD and normal subjects. The amygdala plays an important role in emotional regulation and processing. This is supported by previous studies of AD that identified a number of discriminative functional connectivity associated with the amygdala, including between the left amygdala and the right superior temporal gyrus (Yao et al., 2013) and between the left amygdala and left caudate nucleus (Yao et al., 2013) obtained in this study. In addition, the relatively large weights obtained in this investigation were found to be associated with AD in previous studies, such as the functional connectivity between the right precentral gyrus and right inferior parietal, the supramarginal and angular gyri (Kim and Pan, 2015), the left superior occipital gyrus and right parahippocampal gyrus (Zhou et al., 2015), the left caudate nucleus and right superior parietal gyrus (Wang et al., 2007), the left superior parietal gyrus and left parahippocampal gyrus (Zhou et al., 2015) and the left precuneus and right angular gyrus (Liu et al., 2014).

In the Relief feature selection algorithm, all functional connectivity in the HON-MST were given different weights. Figure 4A shows the results of the average weights of all functional connectivity for each brain region; a number of abnormal brain regions with higher weights were found (Figure 4B), including the left hippocampus, left precuneus, left amygdala, right cuneus, left middle temporal gyrus, right insula, left superior parietal gyrus, left superior frontal gyrus, medial, left caudate nucleus, and right posterior cingulate gyrus. These abnormal brain regions were associated with AD in previous studies. Among them, the weight of the left hippocampus was significantly higher than that of other brain regions. AD is characterized by severe atrophy in the hippocampus, a brain region involved in episodic memory. In AD, the hippocampus is also among the first areas to be damaged, leading to memory impairment and severe cognitive dysfunction. It has been argued that an amnesic syndrome of the hippocampus is an essential core feature for the diagnosis of AD. Previous studies (Zamboni et al., 2013; Aggleton et al., 2016) showed that the left hippocampus plays an important role in AD pathogenesis. The precuneus is part of the brain default network, which is also an important biomarker in AD research (Zamboni et al., 2013). In addition, Grady et al. (2001) found a positive association between left amygdala activity and memory performance in AD patients. They also found that the right cuneus was also an important brain region in AD. Sun et al. (2014) investigated organizational alternations in functional connectivity networks in AD patients using rs-fMRI and identified abnormal brain regions including the left superior parietal gyrus and left caudate nucleus. The other major abnormal brain regions we identified, also in agreement with previous studies, were the right insula (Maxim et al., 2005) and right posterior cingulate gyrus (Zamboni et al., 2013).

The 90 brain regions were divided into 5 modules: frontal, occipital, parietal, subcortical, and temporal. The mean weight values of the functional connectivity between all brain regions in one module and those in the other modules were calculated and used as an interactive weight between the two modules. As depicted in Figure 5A, the interaction weights between frontal and parietal, frontal and temporal, and parietal and occipital were significantly higher than those between the other modules. Moreover, Figure 5B shows the larger weights of discriminative functional connectivity are mainly between temporal and subcutaneous modules, and frontal and parietal modules. Interaction between the frontal and parietal plays an important role in cognitive and memory processing. pathways can lead to memory impairment and executive dysfunction (Grady et al., 2001; Toussaint et al., 2014).

Using magnetic resonance imaging and clinical diagnosis, Grady et al. (2001) showed that interaction between the frontal and temporal lobes play important roles in memory and cognition, and changes in the degree of interaction is one of the most important causes of AD. Figure 5 shows that the interaction weight between the temporal and subcutaneous regions is greater, mainly because of the higher weight of the functional connectivity between the hippocampus and temporal lobe. Notably, this the circuit has an important correlation with AD (Salat et al., 2011). Stam et al. (2006) investigated topographic characteristics of disturbed resting-state networks in AD patients in different frequency bands. They found that the degree of association between the parietal and occipital lobes was related to AD. This is consistent with the present results. Figure 5A shows that the average weights of functional connectivity within the frontal, temporal, and parietal modules are significantly higher than the average weight of the functional connectivity within the other modules. The frontal module is important for the diagnosis of AD (Woodward et al., 2010). The temporal module also participates in spontaneous perception changes (Fraser et al., 2010). Studies have also shown that the parietal module is closely related to AD onset (Jacobs et al., 2012).

Collectively, the results show that the HON-MST can help identify more accurate biomarkers for AD diagnosis, and these findings are consistent with previous studies.

### Classification results

The conventional methods of constructing functional connectivity networks include partial and Pearson correlation functional connectivity networks, both of which are static. To improve the study of functional connectivity dynamics between brain regions, Wee et al. (Wee et al., 2016) used the sliding window method to divide the whole rs-fMRI time series and established a sparse time dynamic network. Using dynamic functional connectivity analysis, they found abundant discriminative information for the diagnosis of MCI and constructed a classification method based on a sparse temporal dynamic network. Chen et al. (2016) constructed a classification method based on a high-order functional connectivity network and found it had high accuracy in diagnosing MCI. The high-order functional connectivity network constructed by Chen et al. is different from the one described in this study. The former used a clustering method to reduce network dimensions, which were related to the number of clusters. In contrast, we constructed a network with fixed dimensions and simplified it with a statistical method.

Table 3 compares the accuracies, specificities, and sensitivities of these classification methods. Existing studies also have differences in feature selection and classification methods. In addition, the investigations were performed using different datasets. In order to avoid effects from different datasets and preprocessing parameter settings, the partial and Pearson correlation functional connectivity networks were constructed with the same dataset and classified using the same feature selection method and classification framework (see Supplemental Text S5 for network construction). The results showed that the performance of the time-varying network constructed by different methods is superior to the static network established with the conventional method. The dynamic functional connectivity network can distinguish AD and normal subjects. Classification performance of the HON-MST is better than those of the high-order functional connectivity network and sparse time dynamic network. Compared with the high-order functional connectivity network proposed by Chen et al., the diagnostic accuracy of the network proposed in this study is improved by about 4%, and the diagnostic accuracy rate of the HON-MST is improved by about 10%. In addition, the classification results show that the HON-MST might be simpler, remove the redundant connectivity effectively, and obtain the key networks, leading to more accurate classification results.

**Figure 6:**
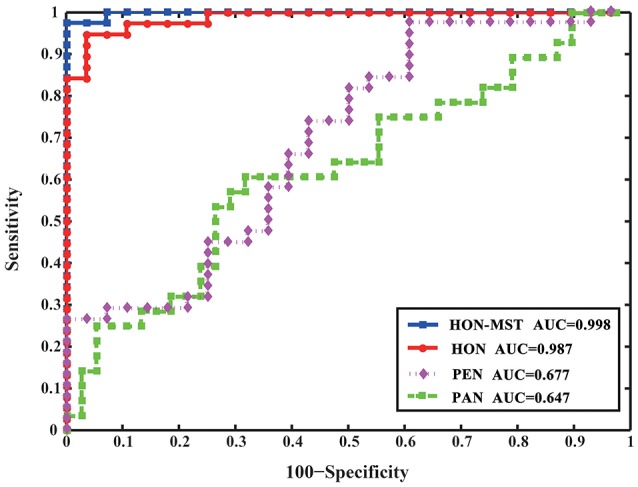
ROC curve of SVM classification of four different methods. HON-MST, minimum spanning tree high-order functional connectivity network; HON, high-order functional connectivity network; PEN, Pearson correlation functional connectivity network; PAN, partial correlation functional connectivity network. PEN and PAN are static networks, HON and HON-MST are dynamic networks. The AUC of the high-order functional connectivity network was 0.987 and the AUC of the HON-MST was 0.998, which were superior to the partial correlation functional connectivity network and Pearson correlation functional connectivity network.

The partial and Pearson correlation functional connectivity networks were constructed using the same dataset and classified using the same feature selection method and classification framework. Figure 6 compares the results of the different classification methods. The AUCs of the high-order functional connectivity network and HON-MST were 0.987 and 0.998, respectively, which are superior to the traditional partial and Pearson correlation functional connectivity networks.

In conclusion, the experimental results suggest that high-order functional connectivity networks and HON-MSTs might reveal more high-level and complex interactions between brain regions, which might significantly improve the accuracy of diagnosing AD compared with conventional methods. At the same time, constructing high-order functional connectivity networks and HON-MSTs may help extract valuable brain regions from the original rs-fMRI time series. In addition, compared with high-order functional connectivity networks, the MST method can effectively reduce network complexity, optimize the network structure, remove redundant and invalid functional connectivity, and identify more efficient key functional connectivity networks. Therefore, the rs-fMRI classification method based on the HON-MST greatly improved AD diagnostic accuracy.

## Methodology

The performance of the classification method depends on the selection of parameters and algorithms such as the MST method, sliding window length *N*, step size *s* of the sliding window movement, weight threshold δ, and correlation coefficient threshold λ in the feature selection and the penalty factor *C* and kernel parameter γ in the SVM model. The choice of these parameters significantly impacts the results. This section describes an analysis of the effect of different parameters and algorithms on the classification results.

### Different MST algorithms

**Figure 7:**
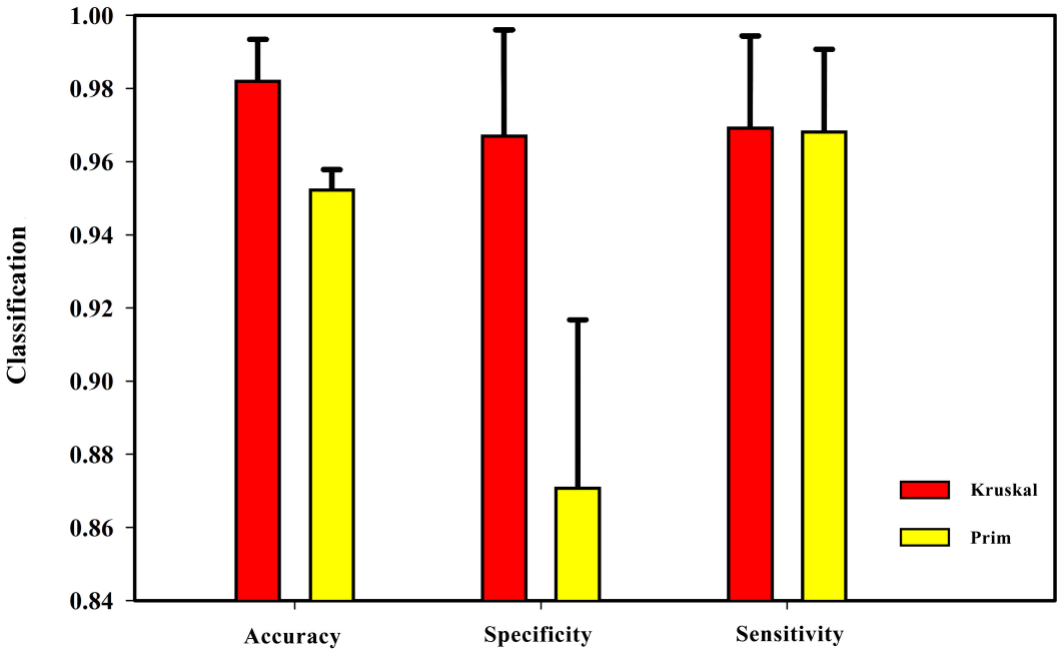
Comparison of Prim algorithm and Kruskal algorithm. The HON-MST based on Prim algorithm and the HON-MST based on Kruskal algorithm were constructed, and the same methods were used for feature selection and classification. The classification accuracy of the HON-MST based on Kruskal algorithm was 98.16%. The classification accuracy of the HON-MST based on Prim algorithm was 95.18%. The classification result of the HON-MST based on Kruskal algorithm was superior to that of the HON-MST based on Prim algorithm.

After obtaining the undirected weighted high-order networks, the networks were simplified by the MST method. There are two well-known algorithms to solve the MST: Prim and Kruskal (see Supplemental Text S4 for details). These two algorithms can solve the undirected weighted graphs of the MST. The Prim algorithm starts at the node of the graph, selecting the nearest node each time until all nodes are united. In contrast, the Kruskal algorithm starts from the edge and always chooses the edge with the least weight. To verify the effect of these two algorithms on the experimental results, the length of the sliding window was set as 60 steps and the step size was 1 TR (2 s). Then, the HON-MSTs based on the Prim and Kruskal algorithms were constructed, and the multi-parameter optimization framework was used for feature selection and classification. Figure 7 shows that the result of the HON-MST based on the Kruskal algorithm is superior. Therefore, this algorithm was used to remove redundant connectivity in the high-order networks.

### Sliding window length

**Figure 8:**
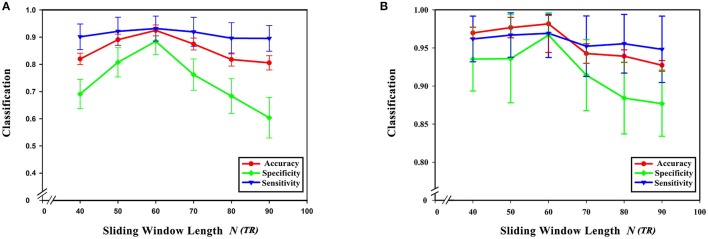
Effect of sliding window length on classification results. **(A)** The effect of different sliding window lengths on the classification results based on high-order functional connectivity network classification method. **(B)** The effect of different sliding window lengths on the classification results based on HON-MST classification method. **(A)** Shows the curve of the accuracy, specificity, and sensitivity of the high-order functional connectivity network classification method of sliding windows with different lengths of 40, 50, 60, 70, 80, and 90 steps, respectively. **(B)** Shows the curve of the accuracy, specificity, and sensitivity of the HON-MST classification method of sliding windows with different lengths of 40, 50, 60, 70, 80, and 90 steps, respectively.

From formula (1) it can be seen that changing the sliding window length alters the number of time windows. At the same time, the number of low-order functional connectivity networks will also be different. In this experiment, the step size was chosen to be 40, 50, 60, 70, 80, and 90 steps. In preprocessing, TR is 2 s (i.e., 1 step is 2 s). The influence of the sliding window length on the classification results was studied while keeping the remaining parameter unchanged. As can be seen from Figure 8, the best results were achieved when the sliding window was 60 s. Diagnostic accuracy is reduced when the sliding window length is too small or large. This can be understood from two aspects. On the one hand, when the value of the sliding window is too large, the number of divided time windows will be smaller, which means that the time-varying characteristics are reduced, which seriously hampers classification accuracy, so the generated networks become unreliable. On the other hand, when the sliding window is too small, similar correlated time series may be divided into different windows, which will increase the number of features extracted from the networks, resulting in more redundant features, making feature selection difficult, and seriously affecting classification accuracy.

**Figure 9:**
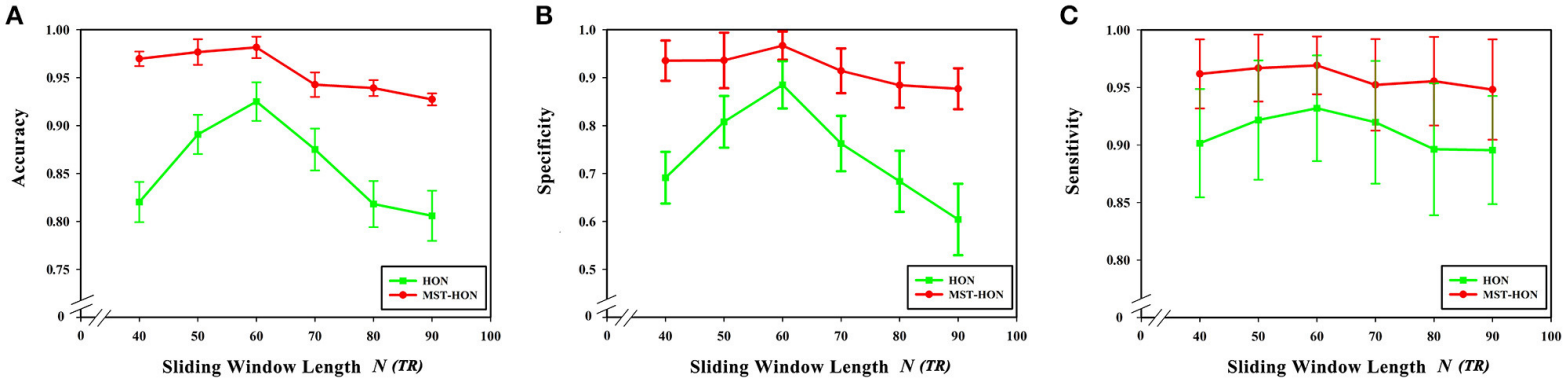
Comparison of the two classification methods of different sliding window lengths. **(A)** Comparison of the accuracy of the two classification methods under different sliding window lengths. **(B)** Comparison of the specificity of the two classification methods under different sliding window lengths. **(C)** Comparison of the sensitivity of the two classification methods under different sliding window lengths. HON, the high-order functional connectivity network; HON-MST, minimum spanning tree high-order functional connectivity networks.

Figures 9A–C compares the accuracies, specificities, and sensitivities of the two methods under different sliding window lengths. The performance of the HON-MST is better than that of the high-order functional connectivity network, regardless of the sliding window length, which indicates that the classification method based on the HON-MST is more accurate and reliable.

### Sliding window step size

**Figure 10:**
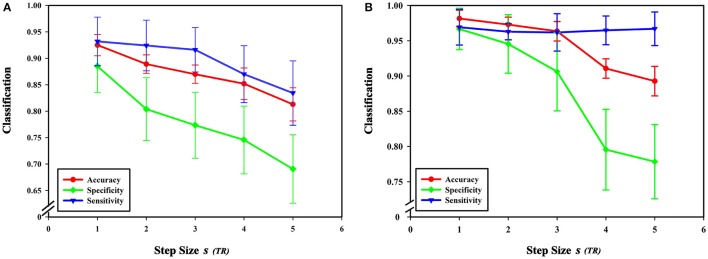
Effect of different step sizes on classification results. **(A)** The effect of different step sizes on the classification results based on high-order functional connectivity network classification method. **(B)** The effect of different step sizes on the classification results based on HON-MST classification method. **(A)** Shows the curve of the accuracy, specificity, and sensitivity of the high-order functional connectivity network classification method of sliding windows with different step sizes of 1 step, 2 steps, 3 steps, 4 steps, and 5 steps, respectively. **(B)** Shows the curve of the accuracy, specificity, and sensitivity of the HON-MST classification method of sliding windows with different step sizes of 1, 2, 3, 4, and 5 steps, respectively.

From formula (1), when the length of the average time series of each brain region is constant, the sliding window length affects the number of time windows, and step size *s* for each sliding window move also impacts the number of time windows. In this experiment, sliding window moving steps of 1, 2, 3, 4, and 5 were selected, while other parameters were kept unchanged, and the length of the sliding window *N* was set to 60 s. The results show that classification accuracy, specificity, and sensitivity were highest when the step size was 1. Figure 10 shows that a larger step size led to poorer classification results, mainly because it reduced the number of time windows. As can be seen from formula (1), the change of the step size has more influence on the number of time windows than does altering the sliding window length. The number of time windows is smaller, which means that the time-varying characteristic is reduced as is classification accuracy, so the generated networks become unreliable.

**Figure 11:**
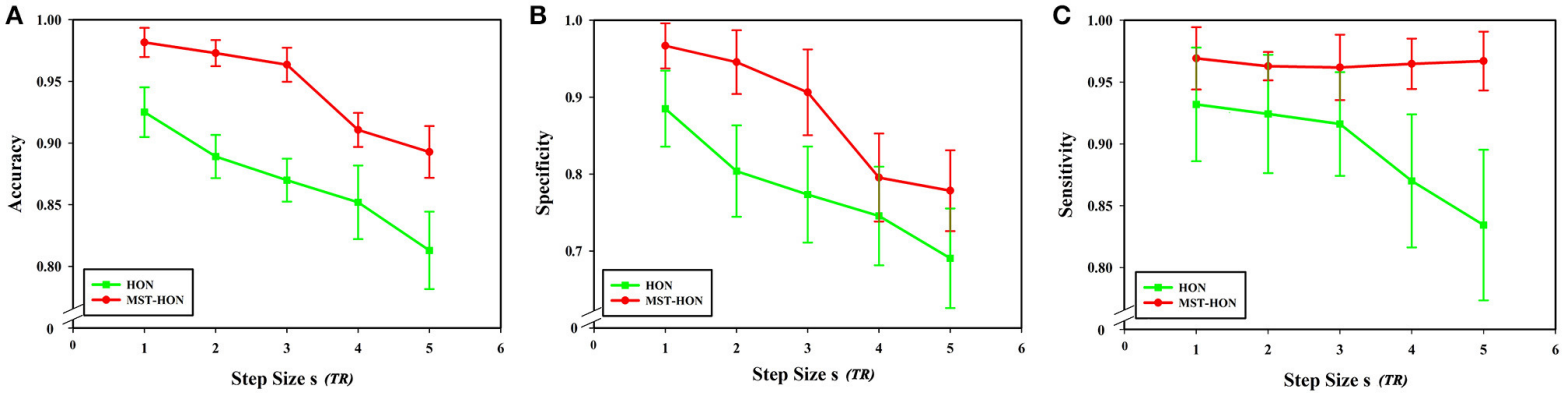
Comparison of the two classification methods of different step sizes. **(A)** Comparison of the accuracy of the two classification methods under different step sizes. **(B)** Comparison of the specificity of the two classification methods under different step sizes. **(C)** Comparison of the sensitivity of the two classification methods under different step sizes. HON, high-order functional connectivity network; HON-MST, minimum spanning tree high-order functional connectivity network.

Figures 11A–C compares the accuracies, specificities, and sensitivities of the two methods under different step sizes. Although the step size is longer, the classification performances of the two methods decline, but the performance of the HON-MST is superior to that of the high-order functional connectivity network.

### Feature selection parameters

In this paper, the multi-parameter optimization framework was used for feature selection and classification. The Relief feature selection method was used for feature selection, the weight of each feature was calculated, the feature was filtered according to the threshold, and the new feature set was obtained. However, this approach could not remove redundant features. The feature set was thus also analyzed by pairwise redundancy analysis, and the Pearson correlation coefficient was calculated to remove features with small weights in the strong correlation feature and obtain the final feature set. Two feature selection parameters are used in this process: the weight threshold δ in the Relief feature selection and the correlation coefficient threshold λ in the redundancy analysis. The choices of these two parameters also impact the classification results.

To select a more accurate weight threshold according to the weighted distribution of all vertices in the network, se selected the weight threshold δ ∈ [1000, 1100, ⋯ , 1600], and the influence of different weight thresholds on the classification results was studied while other parameters were left unchanged.

**Figure 12:**
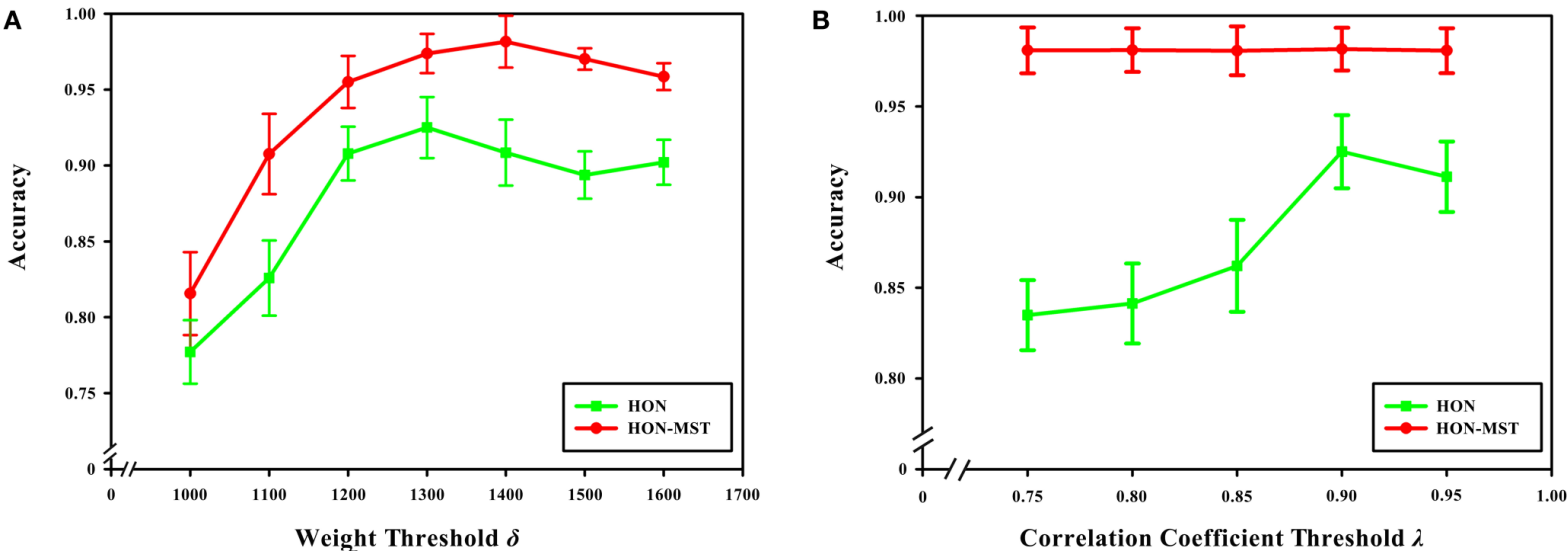
Effect of feature selection parameters on classification results. **(A)** Comparison of the accuracy of the two classification methods under different weight threshold δ. **(B)** Comparison of the accuracy of the two classification methods under different correlation coefficient threshold λ. **(A)** Reflects the effect of different weight thresholds on classification accuracy. When the weight threshold of 1,300, the classification accuracy was the highest in the classification method based on the high-order functional connectivity network. When the weight threshold of 1,400, the classification accuracy was the highest in the classification method based on the HON-MST. **(B)** Reflects the effect of different correlation coefficient thresholds on classification accuracy. When the correlation coefficient threshold of 0.9, the classification accuracy was the highest in the two methods.

With a weight threshold of 1400, the classification accuracy is the highest for the method based on the HON-MST. Figure 12A shows that under the different weight thresholds, the classification method based on the HON-MST is superior to that based on the high-order functional connectivity network. In addition, if the selected weight threshold is small, the features that have less influence are filtered out. Alternatively, if the selected weight threshold is large, the features with larger contributions to the classification are removed. In both cases, classification accuracy decreases.

The Relief feature selection approach is simple and highly efficient. However, a limitation of this method is that it cannot effectively remove redundant features. To overcome this shortcoming, the correlation analysis approach was used to analyze the feature sets extracted by the Relief feature, and then the pairwise Pearson correlation coefficient was calculated to remove redundant features and obtain the final feature set. In this process, a correlation coefficient threshold λ is selected. In this experiment, we chose λ ∈ [0.75, 0.8, ⋯0.95] and studied the influence of different correlation thresholds on the classification results while keeping the other parameters unchanged.

Figure 12B shows that when the correlation coefficient threshold is 0.9, the classification accuracy is highest in the classification method based on the high-order functional connectivity network. This can be understood from two aspects. On one hand, when the threshold is small, redundant features in the feature set cannot be removed, which reduces classification accuracy. On the other hand, when the threshold is large, key features that have a greater impact on the classification result ae removed, which also decreasing the accuracy of the classification. In the classification method based on the HON-MST, the correlation coefficient threshold has little effect on classification accuracy. This is mainly because the goal of pairwise redundancy analysis is to remove redundant features, and the method using the MST can prune the network. For the feature set obtained with the Relief feature selection method, there are few redundant features, so different correlation coefficient thresholds have less influence on the classification accuracy. This also confirms that compared with high-order functional connectivity networks, HON-MSTs have fewer redundant functional connectivity and can be better used for distinguishing between patients with AD and normal subjects.

### SVM parameter optimization

There are two very important parameters in the SVM classification model, namely the penalty factor *C* and kernel parameter γ in the RBF kernel function. These are the key factors affecting SVM performance (Chapelle et al., 2002; Liu et al., 2006). The penalty factor *C* may adjust the range of the confidence interval in the data subspace. When factor *C* is too large, the tolerance for error is low, leading to a tendency for overfitting. Avoiding overfitting is thus a core goal when designing the classifier. In contrast, when *C* is too small, the tolerance for error is high, leading to underfitting. If either over- or underfitting occurs, the generalization ability of the classifier will be reduced, which will affect classification accuracy. The kernel parameter γ is important in the RBF kernel function, which determines the mapping function of the data to the high-dimensional feature space.

**Figure 13:**
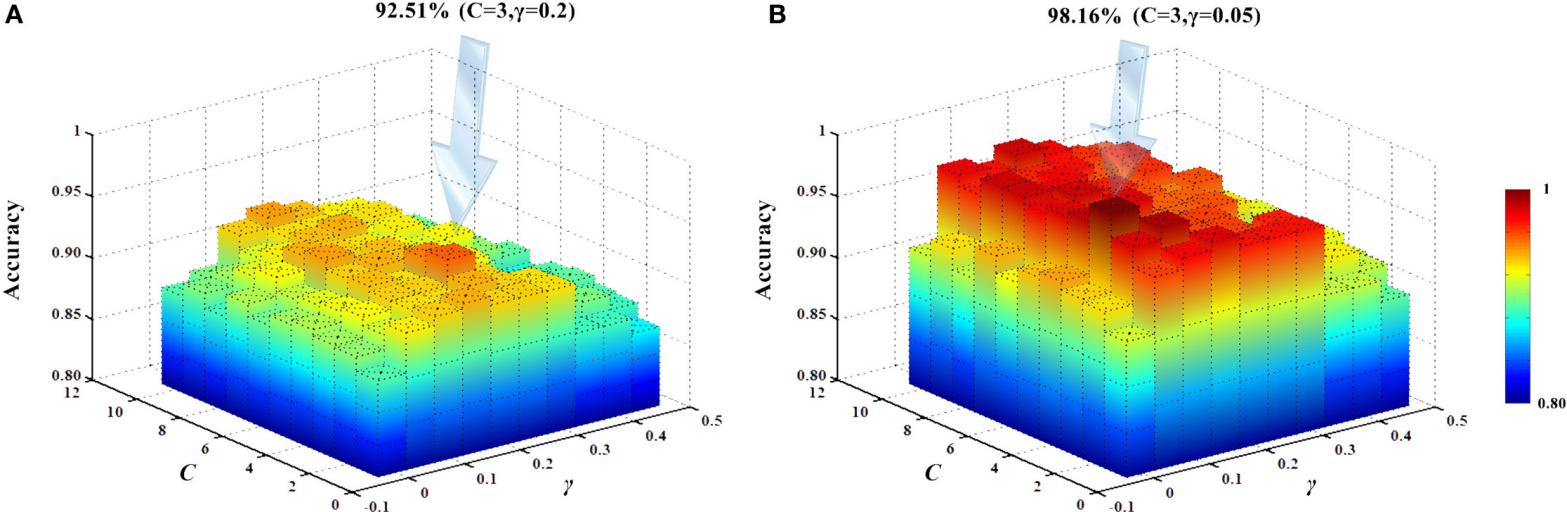
SVM parameter optimization results. **(A)** SVM parameter optimization results of the classification method based on the high-order functional connectivity network. **(B)** SVM parameter optimization results of the classification method based on the HON-MST. **(A)** Shows the change in the accuracy of the classification using different combinations of parameters in the classification method based on the high-order functional connectivity network. Under the current dataset, when *C* = 3 and γ = 0.2, the classification accuracy was the highest. **(B)** Shows the change in the accuracy of the classification using different combinations of parameters in the classification method based on the HON-MST. Under the current dataset, when *C* = 3 and γ = 0.05, the classification accuracy was the highest.

The present study used an SVM classifier based on RBF kernel function. Its performance is determined by the parameters *C* and γ. For different datasets, the default parameter setting using LIBSVM does not give the best classification performance. In other words, different parameter settings should be used for different datasets to obtain the best classification results. We employed the SVM parameter optimization method (Liu et al., 2006) based on a grid search to select different parameter combinations, as follows: *C* ∈ [1, 2, ⋯ , 9, 10], γ ∈ [0, 0.05, 1⋯ , 0.40, 0.45]. Classifier performance with different parameter combinations was evaluated by cross-validation. Figure 13 shows the obtained classification accuracies. Under the current dataset, when *C* = 3 and γ = 0.2, the high-order functional connectivity network classification accuracy was the highest. HON-MST classification accuracy was highest for *C* = 3 and γ = 0.05.

## Limitations of sliding windows method

The sliding window method has been widely applied to discover dynamic changes in neural interactions (Deng et al., 2016; Nomi et al., 2016; Zhu et al., 2016). However, we could not completely avoid the effect of noise signals. This effect is reflected in the sliding window length. A previous study was cautious in setting the length because short windows increase the risk of spurious fluctuations in the observed dynamic functional connectivity, while long windows impede the detection of temporal variations of interest (Preti et al., 2017). Under the premise that we cannot completely remove noise signals, an appropriate sliding window length could limit their effects. Conversely, an unsuitable setting would cause fake connectivity or reduce instantaneity.

Similar studies also demonstrated the importance of sliding window length. Lindquist et al. (2014) indicated that although the sliding window method could explore dynamic changes in functional connectivity, the choice of length was usually arbitrary in previous studies. According to Shakil et al. (2016), the effect on the correlation between the given two time courses, which is a result of the sliding window length, is more serious than other factors including step size, filter parameters, and sampling rate. In addition, Lindquist et al. (2014) stated that “the removal of a highly influential outlying data point will cause a sudden change in the dynamic correlation that may be mistaken for an important aspect of brain connectivity.” Shakil et al. (2016) indicated that this problem was attributable to improper length selection, so it can be avoided when the sliding window is large enough.

Earlier work arbitrarily selected the sliding windows length as 50 steps (Jones et al., 2012; Keilholz et al., 2013). Hindriks et al. (2016) proved that the setting was not convincing because a subset of the real correlations was not observed at these settings. Hindriks and colleagues indicated that it was largely determined by sliding window length if a dynamic function connection could be detected. The authors proposed a corresponding model to appropriately select sliding window length based on the simulation, but it could not be applied because some model parameters were hardly quantified in the real data.

Fortunately, a valuable conclusion on the selection of the length of sliding windows was reached in a previous study. Leonardi et al. (Leonardi and Van De Ville, 2015) found that the minimum window length should be equal to *1/f*_*min*_ to avoid spurious fluctuations, where *f*_*min*_ represents the cut-off frequency of the high-pass filter to remove frequencies in data preprocessing. This has been proved in other similar reports (Kaiser et al., 2016; Lehmann et al., 2017).

In summary, an appropriate window length could ensure the reliability of dynamic connectivity. We obeyed the above standard to set the minimum sliding window length to avoid false connectivity caused by noise to the greatest possible extent. Because there is a lack of consensus on the upper value of the sliding window length, a series of different values were selected to avoid reducing the instantaneity of dynamic connectivity caused by an overly long window. An optimal setting was evaluated according to the classification results.

Although it has some limitations, a recent study reported that the sliding window method with a series of suitable parameters can reveal the real time-varying fluctuation of functional connectivity while avoiding spurious fluctuations (Baczkowski et al., 2017).

## Conclusion

Functional connectivity reflects the interaction between different brain regions, and some functional connectivity are important biomarkers for diagnosing AD. However, existing methods have neglected two aspects. First, previous studies have suggested that the pattern of intrinsic interaction between different brain regions changes over time. If we only study the correlation of the entire rs-fMRI time series, abundant information in each time period would be neglected. On the other hand, functional connectivity between different brain regions are related to each other and may contain important information for diagnosing disease. To overcome the current obstacles, this paper presents an rs-fMRI method of classifying AD based on the HON-MST. The most discriminative functional connectivity of AD patients were also elucidated in this work. The influence of different parameters on classification results was also examined. Compared with existing methods, the results showed the following advantages of HON-MSTs. First, the HON-MST can reflect dynamic functional connectivity that consider time-varying characteristics. Second, the HON-MST can show higher-level and complex interactions between brain regions and enables studying disease-related associations of changes in deeper brain regions. Finally, compared with the conventional method, the rs-fMRI classification method based on the HON-MST greatly improved AD diagnostic accuracy. Compared with the high-order functional connectivity network, the HON-MST has fewer redundant functional connectivity. However, this study has some limitations. The constructed networks reflect the correlation between functional connectivity in the conventional network, but the possibility of false connectivity cannot be ruled out, In addition, associations between two brain regions may be affected by the greater number of functional connectivity. Owing to the large scale of the network, it is not advisable to use the partial correlation method for construction in view of the complexity of the calculation. To solve this problem, we can introduce tools such as hypergraphs for further research and experimental analysis.

## Ethics statement

This manuscript has not been published or presented elsewhere in part or in entirety, and is not under consideration by any another journal. This study was carried out in accordance with the recommendations of the medical ethics committee of Shanxi Province (reference number: 2012013) with written informed consent from all subjects. All subjects have been given written informed consent in accordance with the Declaration of Helsinki. Meanwhile, all the authors have read through the manuscript, approved it for publication. XJ had full access to all of the data in the study and takes responsibility for its integrity and the accuracy of data analysis.

## Author contributions

HG was responsible for the study design and writing the manuscript. LL performed data analysis and statistical processing. YX provided and integrated experimental data. JC supervised the paper. XJ was the heads of the funds and supervised the paper. All authors approved the final version of the manuscript.

### Conflict of interest statement

The authors declare that the research was conducted in the absence of any commercial or financial relationships that could be construed as a potential conflict of interest.

## Footnotes

^1^Neuper and Klimesch *Principles of Brain Dynamics: Global State Interactions*.
