# Supplementary material for: Alzheimer Classification Using a Minimum Spanning Tree of High-Order Functional Network on fMRI Dataset
Source: Front Neurosci. 2017 Dec 1;11:639. doi: 10.3389/fnins.2017.00639 (PMC5717514; doi:10.3389/fnins.2017.00639)
Supplement: Supplementary file 2 [file Presentation2.PDF]

Supplemental Text S2. AAL Template Brain Regions Definition Name and Abbreviation

Node Number	Full Name	Abbreviation
1，2	Precentral gyrus	PreCG
3，4	Superior frontal gyrus, dorsolateral	SFGdor
5，6	Superior frontal gyrus, orbital part	ORBsup
7，8	Middle frontal gyrus	MFG
9，10	Middle frontal gyrus, orbital part	ORBmid
11，12	Inferior frontal gyrus, opercular part	IFGoperc
13，14	Inferior frontal gyrus, triangular part	IFGtriang
15，16	Inferior frontal gyrus, orbital part	ORBinf
17，18	Rolandic operculum	ROL
19，20	Supplementary motor area	SMA
21，22	Olfactory cortex	OLF
23，24	Superior frontal gyrus, medial	SFGmed
25，26	Superior frontal gyrus, medial orbital	ORBsupmed
27，28	Gyrus rectus/straight gyrus	REC
29，30	Insula	INS
31，32	Anterior cingulate and paracingulate gyri	ACG
33，34	Median cingulate and paracingulate gyri	DCG
35，36	Posterior cingulate gyrus	PCG
37，38	Hippocampus	HIP
39，40	Parahippocampal gyrus	PHG
41，42	Amygdala	AMYG
43，44	Calcarine fissure and surrounding cortex	CAL
45，46	Cuneus	CUN
47，48	Lingual gyrus	LING
49，50	Superior occipital gyrus	SOG
51，52	Middle occipital gyrus	MOG
53，54	Inferior occipital gyrus	IOG
55，56	Fusiform gyrus	FFG
57，58	Postcentral gyrus	PoCG
59，60	Superior parietal gyrus	SPG
61，62	Inferior parietal, but supramarginal and angular gyri	IPL
63，64	Supramarginal gyrus	SM
65，66	Angular gyrus	ANG
67，68	Precuneus	PCUN
69，70	Paracentral lobule	PCL
71，72	Caudate nucleus	CAU
73，74	Lenticular nucleus, putamen	PUT
75，76	Lenticular nucleus, pallidum	PAL

77，78	Thalamus	THA
79，80	Heschl gyrus	HES
81，82	Superior temporal gyrus	STG
83，84	Temporal pole: superior temporal gyrus	TPOsup
85，86	Middle temporal gyrus	MTG
87，88	Temporal pole: middle temporal gyrus	TPOmid
89，90	Inferior temporal gyrus	ITG
