# Supplementary material for: Alzheimer Classification Using a Minimum Spanning Tree of High-Order Functional Network on fMRI Dataset
Source: Front Neurosci. 2017 Dec 1;11:639. doi: 10.3389/fnins.2017.00639 (PMC5717514; doi:10.3389/fnins.2017.00639)

Supplemental Text S3. The necessity of dividing the time window

The following figure illustrates the necessity of dividing the time window, taking the functional connectivity between the left calcarine fissure and surrounding cortex and the left superior occipital gyrus as an example. In the traditional rs-fMRI analysis, it is assumed that the correlation coefficient between the two brain regions is a fixed value, that is, the blue dotted line in the figure. However, by dividing the time window, it can be found that the correlation coefficient between the two brain regions is different at each time window, that is, the red line in the graph, indicating that the correlation coefficient between the two brain regions is dynamically changed. This reflects the need to divide time windows and the importance of building dynamic functional connectivity networks.

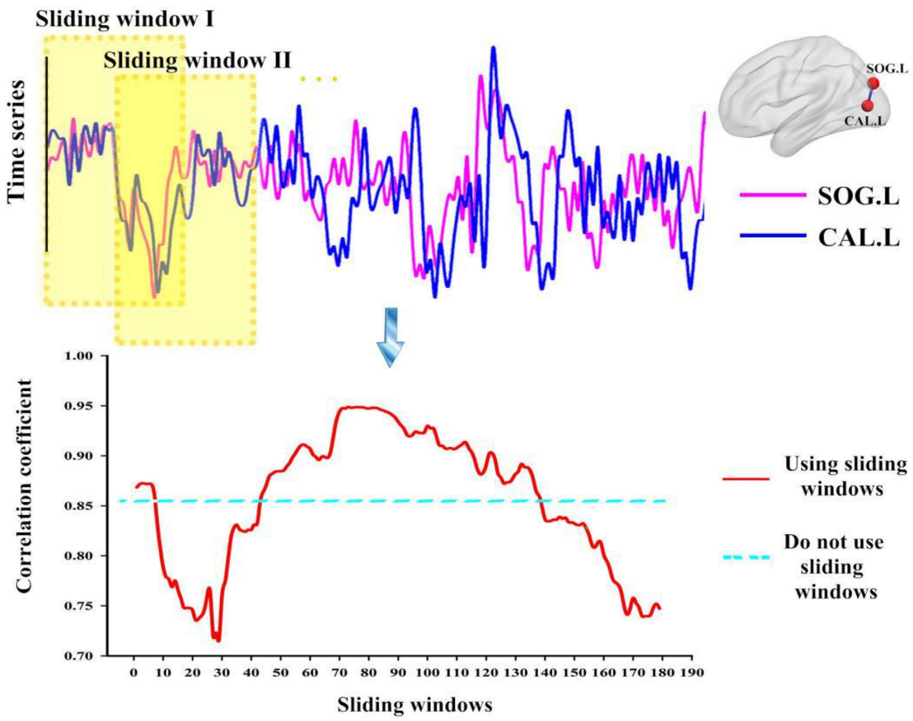

Supplement: Supplementary file 3 [file Presentation3.PDF]
