# Supplementary material for: Alzheimer Classification Using a Minimum Spanning Tree of High-Order Functional Network on fMRI Dataset
Source: Front Neurosci. 2017 Dec 1;11:639. doi: 10.3389/fnins.2017.00639 (PMC5717514; doi:10.3389/fnins.2017.00639)
Supplement: Supplementary file 4 [file Presentation4.PDF]

Supplemental Text S4. Minimum spanning tree algorithms

Minimum spanning tree in weighted graphs - Prim's algorithm[1]

Problem: For a given weighted graph, find a spanning tree with the minimal sum of the weights.

The algorithm is similar to finding the shortest paths in a weighted graphs.

The difference is that we record in the table the length of the current edge, not the length of the path .

Data structures needed:

·A table T with number of rows = number of nodes, and three columns:

$T_{i,1}$ = True if the vertex has been fixed in the tree, False otherwise. This is necessary because the graph is not directed and without this information we may enter a cycle.

$T_{i,2}$ = the length of the edge from the chosen parent (stored in the third column of the table) to the vertex vi,

$T_{i,3}$ = parent of vertex vi

·Adjacency lists

·A priority queue of nodes to be processed.

The priority of each vertex is determined by the weight of edge that links the vertex to its parent. The priority may change if we change the parent.

Algorithm:

1、Initialize first column to False, select a vertex s and store it in the priority queue with priority = 0, set $T_{s,2}$ = 0, $T_{s,3}$ = root

(It does not matter which vertex is chosen, because all nodes have to be in the tree.)

2、While there are nodes in the queue:

a.DeleteMin a vertex v from the queue and set $T_{v,1}$ = True

b.For all adjacent nodes w:

·If $T_{w,1}$ = T do nothing

·If $T_{w,2}$ is empty:

· $T_{w,2}$ = weight of edge (v,w) (stored in the adjacency list)

· $T_{w,3}$ = v (this is the parent)

·Append w in the queue with priority = weight of (v,w)

·If $T_{w,2}$ > weight of (v,w)

·Update $T_{w,2}$ = weight of edge (v,w)

·Update the priority of w(this is done by updating the priority of an element in the queue -decrease Key operation. Complexity $O(logV)$)

·Update $T_{w,3}$ = v

At the end of the algorithm, the tree would be represented in the table with its edges $\{(T_{i,3}, v_i) \mid i = 1, 2, -|V|\}$.

Complexity[2]: $O(|E| log(|V|))$

All edges have to be examined, and in the worst case each edge might cause updating of the priority queue.

If adjacency matrix is used, the complexity would increase to $O(|V|^2)$.

Minimum spanning tree in weighted graphs - Kruskal's algorithm[3]

Kruskal's algorithm works with tree forests and the set of edges.

Algorithm:

1、Initially we build |V| trees consisting of one vertex only - each vertex is a tree of its own.

The edges are stored in a priority queue with priority - the weight.

2、While the number of distinct trees is greater than one:

DeleteMin an edge (u,v) from the priority queue.

a.If u and v belong to one and the same tree, do nothing.

b.If u and v belong to different trees, link the trees by the edge

Implementation

The algorithm is implemented using operations on disjoint sets. All the vertices are grouped into sets corresponding to the currently built trees. Since each vertex appears in one tree only, the sets are disjoint.

Two set operations are used:

a.comparison:

Each vertex is associated with the disjoint set where it belongs.

To find out whether two nodes are in the same tree, we need to find out if their disjoint sets are the same.

b.union:

When we link the trees, the combine the two disjoint sets to form one set- corresponding to the new tree

Complexity of Kruskal's algorithm[2]:

A detailed analysis will show O(|V|) + O(|E|log(|E|)) + O(|E|log(|V|)).

a.We need O(|V|) operations to build the initial forest with |V| trees each containing one node.

b.The edges are stored in a priority queue and each time the smallest edge is retrieved, hence we need O(|E|log(|E|)) operations to process the edges.

c.Finally, the disjoint set operations are implemented by a tree with |V| nodes, hence we need O(|E|log(|V|)) operations (a comparison is performed for each edge in the worst case).

Disregarding the lower term O(|V|) we get O(|E|(log(|V|) + log(|E|)).

At the worst case |E| = O(|V|2). Hence log(|E|) = O(log(|V|2)) = O(2log(|V|)) = O(log(|V|).

Thus we get complexity O(|E|log(|V|)).

On the other hand, |V| = O(|E|), hence we can reduce the complexity expression to O(|E|log(|E|)).

Reference

1. Prim, R.C., *Shortest connection networks and some generalizations.* Bell System Technical Journal, 1957. **36**(6): p. 1389-1401.
2. Weiss, M.A., *Data structures and algorithm analysis in java.* 2011. **253**(6564): p. 1073.
3. Kruskal, J.B., *On the Shortest Spanning Subtree of a Graph and the Traveling Salesman Problem.* Proceedings of the American Mathematical Society, 1956. **7**(1): p. 48-50.
