# Supplementary material for: Alzheimer Classification Using a Minimum Spanning Tree of High-Order Functional Network on fMRI Dataset
Source: Front Neurosci. 2017 Dec 1;11:639. doi: 10.3389/fnins.2017.00639 (PMC5717514; doi:10.3389/fnins.2017.00639)
Supplement: Supplementary file 5 [file Presentation5.PDF]

Supplemental Text S5. The traditional methods of constructing functional connectivity networks

Pearson correlation method

Node definition: In the current study, a prior atlas of automated anatomical labeling was used to define the nodes. The brain was divided into 90 regions (45 for each hemisphere), with each region representing a node in the network. The mean time series for all voxels in each region was calculated as part of the time series of the corresponding node.

Edge definition: We used Pearson correlation coefficient to calculate the correlation coefficient of average time series between any two nodes. Before the time series of nodes, we used multiple linear regression analysis with head motion profiles, estimated from the image realignment, to correct the effects of head motion. The residuals were then used to compute the partial correlation, producing a 90×90 correlation matrix.

Partial correlation method

Node definition: In the current study, a prior atlas of automated anatomical labeling was used to define the nodes. The brain was divided into 90 regions (45 for each hemisphere), with each region representing a node in the network. The mean time series for all voxels in each region was calculated as part of the time series of the corresponding node.

Edge definition: We computed partial correlation coefficients as edges in the network. Before the time series of nodes, we used multiple linear regression analysis with head motion profiles, estimated from the image realignment, to correct the effects of head motion. The residuals were then used to compute the partial correlation, producing a 90×90 correlation matrix.
