# Supplementary material for: Alzheimer Classification Using a Minimum Spanning Tree of High-Order Functional Network on fMRI Dataset
Source: Front Neurosci. 2017 Dec 1;11:639. doi: 10.3389/fnins.2017.00639 (PMC5717514; doi:10.3389/fnins.2017.00639)

Figure 1. Time window segmentation diagram. *M* represents the length of the fMRI time series; *N* represents the length of the sliding window; *s* represents the step size of the sliding window every movement.

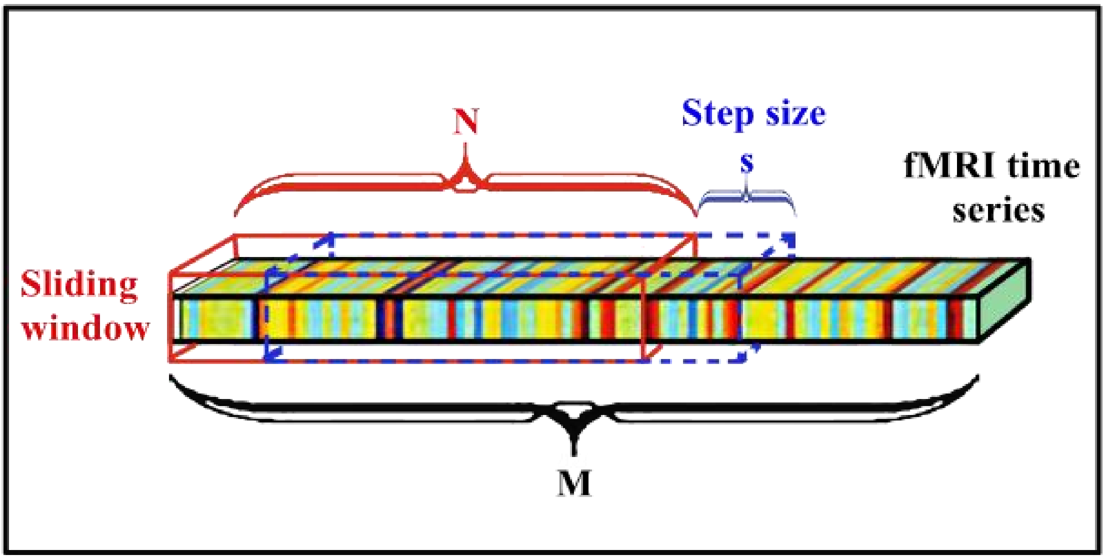

Supplement: Supplementary file 6 [file Image1.PDF]
