# Supplementary material for: Alzheimer Classification Using a Minimum Spanning Tree of High-Order Functional Network on fMRI Dataset
Source: Front Neurosci. 2017 Dec 1;11:639. doi: 10.3389/fnins.2017.00639 (PMC5717514; doi:10.3389/fnins.2017.00639)
Supplement: Supplementary file 7 [file Table1.DOCX]

**Table 1 Results of multiple** **linear regression analysis between network properties and confounding variables**

| Influencing factors | Gender | Age | Educational Attainments |
| --- | --- | --- | --- |
| Adj. R_sqr_ | 0.086 | 0.058 | -0.031 |
| P | 0.391 | 0.652 | 0.565 |

The range of age is 17–51 years. Optional values of gender are male and female. Optional values of educational attainments are illiteracy, primary school, junior high school, senior high school, junior college, college, graduate degree and above. Adj. R_sqr_, adjusted R square. P，significance.
